# Estimation of species divergence times in presence of cross-species gene flow

**DOI:** 10.1093/sysbio/syad015

**Published:** 2023-03-24

**Authors:** George P Tiley, Tomáš Flouri, Xiyun Jiao, Jelmer W Poelstra, Bo Xu, Tianqi Zhu, Bruce Rannala, Anne D Yoder, Ziheng Yang

**Affiliations:** Department of Biology, Duke University, Durham, NC, USA; Department of Genetics, Evolution and Environment, University College London, London, UK; Department of Genetics, Evolution and Environment, University College London, London, UK; Department of Statistics and Data Science, China Southern University of Science and Technology, Shenzhen, Guangdong, China; Department of Biology, Duke University, Durham, NC, USA; Beijing Institute of Genomics, Chinese Academy of Sciences, Beijing 100101, China; National Center for Mathematics and Interdisciplinary Sciences, Academy of Mathematics and Systems Science, Chinese Academy of Sciences, China; Key Laboratory of Random Complex Structures and Data Science, Academy of Mathematics and Systems Science, Chinese Academy of Sciences, China; Department of Evolution and Ecology, University of California, Davis, Davis, CA, USA; Department of Biology, Duke University, Durham, NC, USA; Department of Genetics, Evolution and Environment, University College London, London, UK

## Abstract

Cross-species introgression can have significant impacts on phylogenomic reconstruction of species divergence events. Here, we used simulations to show how the presence of even a small amount of introgression can bias divergence time estimates when gene flow is ignored in the analysis. Using advances in analytical methods under the multispecies coalescent (MSC) model, we demonstrate that by accounting for incomplete lineage sorting and introgression using large phylogenomic data sets this problem can be avoided. The multispecies-coalescent-with-introgression (MSci) model is capable of accurately estimating both divergence times and ancestral effective population sizes, even when only a single diploid individual per species is sampled. We characterize some general expectations for biases in divergence time estimation under three different scenarios: 1) introgression between sister species, 2) introgression between non-sister species, and 3) introgression from an unsampled (i.e., ghost) outgroup lineage. We also conducted simulations under the isolation-with-migration (IM) model and found that the MSci model assuming episodic gene flow was able to accurately estimate species divergence times despite high levels of continuous gene flow. We estimated divergence times under the MSC and MSci models from two published empirical datasets with previous evidence of introgression, one of 372 target-enrichment loci from baobabs (*Adansonia*), and another of 1000 transcriptome loci from 14 species of the tomato relative, *Jaltomata*. The empirical analyses not only confirm our findings from simulations, demonstrating that the MSci model can reliably estimate divergence times but also show that divergence time estimation under the MSC can be robust to the presence of small amounts of introgression in empirical datasets with extensive taxon sampling. [divergence time; gene flow; hybridization; introgression; MSci model; multispecies coalescent]

Divergence time estimation has been an area of rich investigation in evolutionary biology and instrumental in revealing the mode and tempo of evolution across the tree of life. Sophisticated relaxed-clock methods (Thorne et al. 1998; Drummond et al. 2006; Lepage et al. 2007; Rannala and Yang 2007) exist that can leverage multiple marginal prior distributions that represent external fossil (Benton and Donoghue 2007) or geological (De Baets et al. 2016) calibrations while allowing variation in the rate of molecular sequence evolution among lineages. These relaxed-clock methods make the critical assumption, however, that all loci share a single species tree topology and the same set of divergence times. Evolutionary histories of individual loci henceforth referred to as “gene trees” (regardless of whether a locus is an annotated functional gene), may conflict with the species tree (Maddison 1997) for which divergence times are being estimated. A common source of conflict between gene trees and species trees is incomplete lineage sorting (ILS) or delayed coalescent, which occurs when sequences from different species do not coalesce in the youngest ancestral species but coalesce in more ancient ancestors (Hudson 1983; Pamilo and Nei 1988; Degnan and Rosenberg 2009). ILS can be particularly common if the time between speciation events is short and if the ancestral population size is large, and is prevalent across diverse organismal groups at both recent and ancient timescales (Angelis and dos Reis 2015). Although ILS has largely been studied in the context of species tree estimation (Degnan and Rosenberg 2009), accounting for ILS explicitly with the multispecies coalescent model (MSC; Rannala and Yang 2003) can also improve the estimation of substitution rates and divergence times when gene tree discordance is high (Angelis and dos Reis 2015; Ogilvie et al. 2017; Stange et al. 2018).

Episodic gene flow between species after they have diverged (introgression) is also known to affect species divergence times but is perhaps less well understood than ILS. Advances in statistical tests for introgression (Green et al. 2010; Pease and Hahn 2015; Blischak et al. 2018; Ji et al. 2022) and phylogenetic network estimation (Solís-Lemus and Ané 2016; Wen et al. 2016; Zhang et al. 2018a) coupled with contemporary phylogenomic datasets have characterized signals of introgression in many non-model groups across mammals (Nilsson et al. 2018; Poelstra et al. 2021), insects (Edelman et al. 2019; Hundsdoerfer et al. 2019), birds (Oswald et al. 2019; Zamudio-Beltran et al. 2020), and squamates (Schield et al. 2017; Barley et al. 2019). Introgression is especially common in plants—which are notorious for allopolyploid speciation (Barker et al. 2016)—both within genera (Crowl et al. 2017; Wu et al. 2018; Karimi et al. 2020) and at deeper phylogenetic levels (Stull et al. 2020; Morales-Briones et al. 2021). Full-likelihood MSC methods, such as StarBEAST3 (Douglas et al. 2022) and bpp (Flouri et al. 2022) can use these phylogenomic datasets to estimate divergence times among species with a strict or relaxed molecular clock while accommodating for the variation in gene trees at individual loci. It is also possible to estimate the timing of introgression and the proportion of introgressed loci under the MSC-with-introgression (MSci) model (Flouri et al. 2020). While computationally demanding, recent bpp implementations of the MSC with and without gene flow have been successfully applied to data of 10,000 loci for species trees with a small number of tips (e.g., Shi and Yang 2018; Thawornwattana et al. 2022).

Here, we examine the impacts of introgression on divergence time estimation using the MSci model. In previous studies, post-divergence gene flow between sister species, if ignored, was found to lead to underestimation of the divergence time between those species while all other nodes in the species tree are not affected (Leache et al. 2014; Barley et al. 2018). Gene flow between non-sister species also causes underestimation of their divergence times (Leache et al. 2014; Tonzo et al. 2021). In this study, we not only revisit these scenarios but also use simulation to investigate the effect of gene flow from an unsampled outgroup species. The prevalence of unsampled or “ghost” lineages is well-documented in human demographic history (Excoffier et al. 2013) and in cases of domestication (Wang et al. 2020). Because the MSci model represents gene flow as an episodic introgression event, which may not be realistic in some situations, we also simulated data under the isolation-with-migration (IM; Nielsen and Wakeley 2001) model with continuous gene flow to examine whether the MSci analysis is capable of accurately estimating species divergence times despite the misspecification of the mode of gene flow. To examine whether the results obtained from the simulation study apply to real data analysis, we estimated divergence times under the MSC and MSci models in empirical datasets from baobabs (*Adansonia* Malvaceae) and *Jaltomata* (Solanaceae) that have complex evolutionary histories with evidence of ancient introgression between non-sister lineages. We find that the assumed model of gene flow—continuous versus episodic—can have significant and differential impacts on divergence time estimates and that the MSci model was capable of accurately estimating divergence times in both cases.

## Materials and Methods

### Simulating Gene Trees and Sequence Alignments Under MSC Models with Introgression

**Figure 1.**
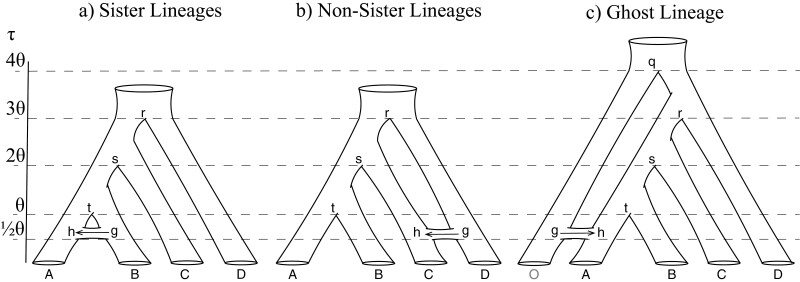
Species networks used for simulations. a) Gene flow between sister lineages: species A and B diverged at time *τ*_*t*_, and introgression occurred from B into A at time *τ*_*h*_. b) Gene flow between non-sister lineages (from species D into C at time *τ*_*h*_. c) Gene flow from an unsampled ghost lineage O (shown in gray) into species A. Divergence time (τ) is given in units of population size (θ). Population size is constant among all branches. Node names are shown with lower-case letters. The direction of introgression is from node g to h, indicated by the arrow. Simulations under the IM model use the same species trees, but with migration occurring after species divergence at the constant rate of *M* = *Nm* migrants per generation.

**Figure 2.**
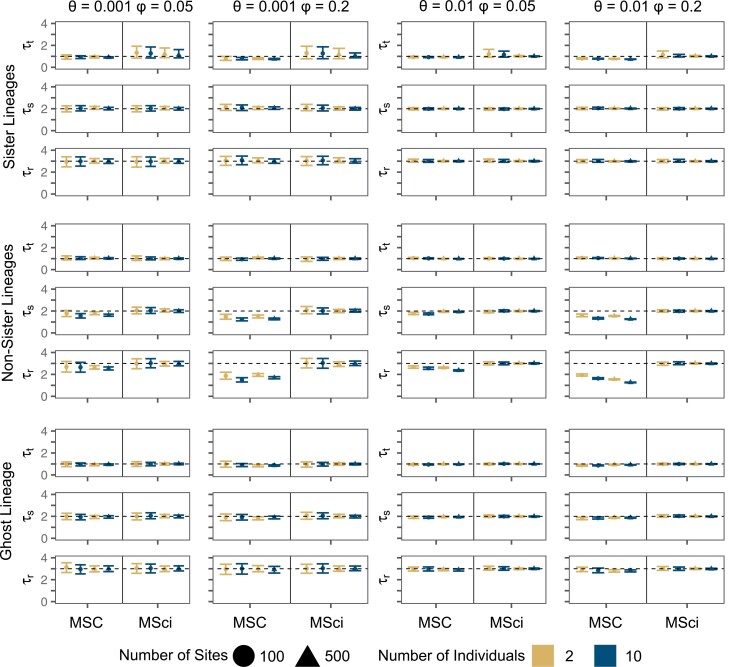
divergence time estimates for speciation nodes simulated under the MSci Model. True values are shown with a dashed horizontal line. Points are posterior means and error bars are 95% highest posterior density (HPD) credible intervals (CIs), both averaged over 10 replicates. The *y*-axis scale is $\times 10^{-3}$ and $\times 10^{-2}$ for when θ=0.001 and θ=0.01, respectively.

**Figure 3.**
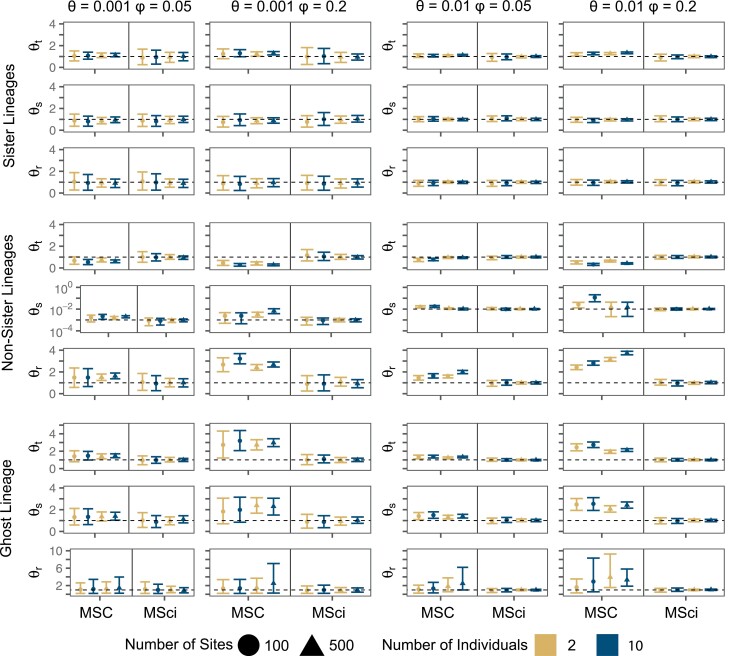
Population size estimates for speciation nodes simulated under the MSci model. True values are shown with a dashed horizontal line. Points are posterior means and error bars are 95% HPD CIs, averaged over 10 replicates. $\theta_{s}$ for the non-sister scenario is shown on the log10 scale. The *y*-axis scale is $\times 10^{-3}$ and $\times 10^{-2}$ for when θ=0.001 and θ=0.01, respectively.

We simulated sequence data under the MSci model under three scenarios: 1) post-divergence introgression between two sister lineages (Fig. 1a), 2) historical introgression involving two non-sister lineages (Fig. 1b), and 3) introgression from an outgroup ghost lineage that has gone extinct or is unsampled (Fig. 1c). The simulate option of bpp v4.1.4 (Flouri et al. 2020) was used to simulate gene trees with coalescent times (branch lengths) under the MSci model and to evolve sequences on the gene tree. Our simulation concerns the analysis of recently diverged species. Thus, the Jukes–Cantor model of nucleotide substitution (Jukes and Cantor 1969) was used to simulate and analyze data, assuming a strict molecular clock and no rate variation among sites or loci.

Each dataset consists of 1000 loci. We consider the effects of several factors: 1) sequence length, 2) the number of sequences sampled per species, 3) introgression probability (φ or the proportion of immigrants in the receiving population at the time of introgression event), and 4) the mutation-scaled population size (θ) measured by the expected number of substitutions per site between two sequences sampled from the species. We used two sequence lengths: 100 base pairs (bps) and 500 bps. Many early single-end RADseq experiments generated loci not much longer than 100 bp (Rubin et al. 2012, Eaton and Ree 2013). Target-enrichment methods (Faircloth et al. 2012; Lemmon et al. 2012; Johnson et al. 2019; Breinholt et al. 2021) or recent paired-end RADseq protocols (e.g., Ali et al. 2016) can generate many loci of at least 500 bp in length. We used two values of θ: 0.001 and 0.01. As divergence times (τ) are proportional to θ in our simulation, the different values of θ mimic the use of loci with different mutation rates (e.g., ultraconserved elements versus introns). In conjunction with sequence length, mutation rate or θ affects the amount of phylogenetic information in the dataset. For example, a θ of 0.001 implies on average one nucleotide difference every 1000 bp between two sequences from the species, and if the sequence is very short (at 100 bps), there may be many invariable and therefore uninformative loci. At the high mutation rate (θ = 0.01) and with longer loci (500 bp), five nucleotide differences will be expected between two randomly sampled sequences from the species, so that the data will be more informative for estimating θ.

Because the MSC and MSci models can accommodate multiple individuals per species to estimate population genetic parameters, we simulated data with either 2 or 10 haploid sequences. Two haploid sequences are similar to one diploid individual, which can be analytically phased (Gronau et al. 2011; Huang et al. 2022a). We expect that sampling many individuals will not greatly improve the estimation of species divergence times, as two haploid sequences or one diploid individual is sufficient to estimate the divergence time (Wakeley 2009; Huang et al. 2020). Simulations were also used to explore the effects of increasing introgression probabilities on divergence times and population sizes. We used φ of 0.05 or 0.2 to represent low and moderate levels of introgression. Because gene flow is in many scenarios likely not a single episodic event, instead being a continuous or steadily diminishing process, we repeated all simulations under an IM model. All simulation conditions except for the mode of gene flow were the same as under the MSci model, except that under the IM model, we used a migration rate (*M* = *Nm*) of either 0.1 or 1.0 migrant individuals per generation. *Nm* = 0.1 may represent a moderate rate of migration between species, while *Nm* = 1 is an extremely high rate, perhaps typical for migration between populations of the same species.

Simulated data were analyzed using bpp v4.1.4 to estimate parameters in the MSC or MSci models. We collected 200,000 posterior samples after a burn-in of 20,000 MCMC iterations, sampling every two iterations. The prior on θ was assigned the inverse gamma prior IG(*α, β*) with *α* = 3 and with *β* chosen so that the prior mean *β*/(*α* – 1) equals the true value. The age of the root (*τ*_*r*_) was assigned an inverse gamma prior IG(*α, β*) with *α* = 3 and prior mean equal to the true value. Given *τ*_*r*_, the other species’ divergence times (*τ*) follow a uniform Dirichlet distribution (Yang and Rannala 2010, Equation 2). Note that the inverse gamma is a heavy-tailed distribution and *α* = 3 means a diffuse prior. For the MSci model, the prior beta(1,1) is assigned on φ, which is equivalent to the uniform U(0, 1). Pilot MCMC runs were used to assess MCMC settings, with convergence assessed by the consistency between runs. Additional scrutiny was given to seemingly aberrant runs.

Median running time for analyzing one replicate dataset using one thread ranges from 3 h for small datasets (with two sequences per species, 100 bps in the sequence, and low mutation rate *θ* = 0.001) to 120 h for large datasets (with 10 sequences per species, 500 bps in the sequence and the high mutation rate *θ* = 0.01) (Table S1).

### Impacts of Introgression on Divergence Time Estimation in Empirical Data

Two published datasets with strong evidence for historical introgression were used to estimate divergence times under the MSC and MSci models. Both systems are reasonably complex and allow us to evaluate whether results from simulations based on small species trees appear to apply to larger species trees with more tips and potentially multiple introgression events. First, we estimated divergence times for *Adansonia* (Karimi et al. 2020), using 372 target-enrichment loci generated from a custom probe set developed by the original authors (Karimi et al. 2020). Previous analyses identified a single ancient introgression event in *Adansonia* with SNaQ (Solís-Lemus and Ané 2016) as implemented in PhyloNetworks v0.12.0 (Solís-Lemus et al. 2017), but the exact placement of the reticulation edge was sensitive to outgroup choice and sequence assembly methods*. Adansonia* is notable for its endemic Malagasy radiation and reproduce either in the dry season with bat- and lemur-pollinated flowers (section Brevitubae) or in the wet season with generally moth-pollinated flowers (section Longitubae; Baum 1995). Previous analyses suggested ancient introgression in baobabs occurred 1) between mainland African *Adansonia digitata* and the endemic Malagasy section Brevitubae or 2) was restricted within the paraphyletic endemic Malagasy section Longitubae (Karimi et al. 2020). Based on species distributions, morphology, and the *D-*statistic, the authors determined that ancient introgression within section Longitubae was most likely (Karimi et al. 2020). Using the backbone topology from Karimi et al. (2020) and the two introgression hypotheses, we estimated τ and θ under the MSC as well as φ under the MSci using bpp v4.1.4 (Flouri et al. 2020). We estimated parameters in the MSci model assuming either or both of the introgression events. Divergence times were also estimated when introgression was ignored under the MSC. Each analysis used four independent runs that recorded 10,000 posterior samples, sampling every 200 iterations after a burn-in of 200,000 generations. Running time using one thread was 130–150 h, depending on the number of introgression events assumed in the MSci model. We compared the MSC and MSci models using marginal likelihood values calculated with bpp v4.1.4 (Rannala and Yang 2017) and stepping-stone sampling (Xie et al. 2011) using 24 steps with the R package bppr (https://github.com/dosreislab/bppr). Divergence times in substitutions per site were rescaled to absolute times by using a node calibration, with the divergence between *Scleronema* and *Adansonia* fixed at 18.2 Ma (Marinho et al. 2014), and using a rate calibration (see Supplementary Methods: Calibrating Divergence Times).

The second dataset consists of 1000 alignments assembled from transcriptomes of 14 *Jaltomata* species (Wu et al. 2018) and their outgroup *Solanum lycopersicum*. *Jaltomata* are notable as a recent radiation in the Neotropics, especially the Andes, that has clades with distinct fruit color (Miller et al. 2011). Analyses with the *D-*statistic have suggested ancient introgression between the early diverging purple-fruited clade and the green- and orange-fruited clades (Wu et al. 2018). Because the current version of bpp requires an *a priori* specification of the full MSci model, including the species tree topology, the number and direction of introgression events, and the species involved in introgression, we re-analyzed the transcriptome data and estimated a phylogenetic network. We first estimated maximum likelihood (ML) gene trees from 6431 one-to-one ortholog alignments using IQ-TREE v1.6.9 (Nguyen et al. 2015) with the best substitution model selected using ModelFinder (Kalyaanamoorthy et al. 2017). We estimated a species tree with ASTRAL-III v5.6.3 (Zhang et al. 2018b), and used it as the starting tree for a network search with SNaQ (Solís-Lemus and Ané 2016), implemented in PhyloNetworks (Solís-Lemus et al. 2017). We performed searches that allowed between zero and seven reticulation events and allowed 30 independent optimizations per search. The optimal number of reticulations was determined using slope heuristics (Solís-Lemus and Ané 2016).

bpp v4.1.4 was then used to estimate parameters under the MSC and MSci models assuming the estimated species tree and network, respectively. To reduce the computation, we used only 1000 orthologous alignments sampled at random from the original data set of 6431 alignments (Wu et al. 2018). The estimated introgression model includes an introgression from a ghost lineage (see “Results *Analyses of Empirical Data*”), which could be due to technical artifacts from the network-inference methods. We calculated marginal likelihoods to compare models with and without introgression, in particular the introgression involving a ghost lineage. We used stepping-stone sampling with 24 steps, and at each step, collected 10,000 posterior samples, saving every 200 iterations after a burn-in of 500,000 generations. For our best network hypothesis and the species tree, we estimated divergence times using the MSci and MSC, respectively. Both analyses used four independent runs, using 500,000 MCMC iterations as burn-in and then taking 10,000 samples, sampling every 200 iterations. Running time using eight threads ranged from 70 to 90 h. Divergence times were rescaled from substitutions per site to absolute time by assuming a divergence time of 17 Ma ago between *Solanum* and *Jaltomata* (Sarkinen et al. 2013) and using a rate calibrations (Supplementary Methods: Calibrating Divergence Times).

## Results

### Simulated Data with MSci

#### Divergence times

When data were simulated with introgression between sister species A and B (Fig. 1a), the divergence time between those species $\left(\tau_{t}\right)$ was underestimated under the MSC ignoring gene flow, regardless of the sequence length or the number of individuals sampled per species (Fig. 2 and Supplementary Fig. S1). The bias was slightly larger at the high introgression rate (φ=0.2) than at the low rate (φ=0.05) but overall the bias was small. The underestimation of $\tau_{t}$ is clearly due to recent sequence divergences between species A and B caused by gene flow. Note that sequences from two species cannot coalesce until they reach the common ancestral species so that sequence divergence must be older than species divergence. As a result, the estimate of species divergence time tends to be dominated by the smallest sequence divergence between the species. Older divergence times between species not involved in the introgression ($\tau_{s}$ and $\tau_{r}$) were correctly estimated under MSC. The results are consistent with the observation of Huang et al. (2022b) that the impact of gene flow tends to be local, affecting the divergence times (and population sizes) of species involved in gene flow. Under the MSci model, estimates of $\tau_{t}$ for the divergence between A and B were much less certain, with wider 95% highest posterior density (HPD) credibility intervals (CI), than under the MSC, but the estimates (posterior means) did not have the bias of the MSC estimates (Supplementary Figs. S1 and S2). Using longer sequences (500 bps v. 100 bps) improved the posterior estimates of all divergence times far more than sampling more sequences per species (10 sequences vs. 2) (Fig. 2 and Supplementary Fig. S2).

In the case of introgression involving non-sister species from D into C (Fig. 1b), the divergence time $\left(\tau_{r}\right)$ for the two species involved in introgression was underestimated under the MSC, with the bias to be greater at the higher introgression rate (*φ* = 0.2 vs. 0.05) and in more informative datasets, that is, at the high mutation rate of *θ* = 0.01 (versus 0.001), with 10 instead of 2 individuals sampled per species, and with longer sequences of 500 (instead of 100) sites (Fig. 2 and Supplementary Fig. S3). The age $\tau_{s}$ was underestimated under the MSC as well, although the bias was smaller than for $\tau_{r}$, while $\tau_{t}$ did not appear to be affected at all. The results are consistent with previous studies which showed underestimation of species divergence times in presence of introgression (Leache et al. 2014; Poelstra et al. 2021). The MSci model produced good estimates of all divergence times, with the 95% CIs including the true values and with the CIs becoming narrow with the increase of the data size (Fig. 2 and Supplementary Fig. S4).

For the scenario of introgression from an unsampled ghost lineage O into an ingroup species A (Fig. 1c), divergence times $\tau_{t},\tau_{s},\;\text{and}\;\tau_{r}$ were slightly underestimated under the MSC model ignoring gene flow, but the bias was very small (Fig. 2 and Supplementary Fig. S5). This lack of effects may be explained by the fact that estimates of species divergence times are dominated by the smallest between-species sequence divergences, which are identical between the true introgression model and the fitting MSC model. For example, $\tau_{t}$ in MSC is informed by the shortest sequence distance (or coalescent time) between species A and B in the data, which is determined by the parameter $\tau_{t}$ in the true MSci model. The O→A introgression will generate some A sequences that look very different from the B sequences, but will not generate A sequences that look unusually similar to the B sequences. The same argument applies to $\tau_{s}$ and $\tau_{r}$. Finally, the MSci model was able to correctly estimate all divergence times at all levels of sequence divergence, sequence length, and the number of individuals (Fig. 2 and Supplementary Fig. S6), and there was little difference in time estimates under the MSci at the low and high introgression rates. The time of introgression $\left(\tau_{g}\right)$ was well estimated too (Supplementary Fig. S6). Even though no extant sequence data were available from the ghost lineage, the model was able to accurately estimate the divergence and introgression times on the species tree. Note that the MSci analysis assumed the correct model, with the introgression from the ghost species specified in the model, even though no sequence data from the ghost were used.

#### Population sizes

For the sister-species introgression, the MSC model ignoring gene flow produced slight overestimates of $\theta_{t}$ for the common ancestor of species A and B in informative datasets (at the high mutation rate and/or with 10 sequences per species) (Fig. 3 and Supplementary Fig. S7). This is related to the underestimation of the divergence time $\left(\tau_{t}\right)$ between the two species, as $\theta_{t}$ and $\tau_{t}$ are expected to be negatively correlated (Burgess and Yang 2008). Population sizes for other species did not show obvious biases. The MSci model produced overall good estimates, with no apparent bias (Supplementary Fig. S8). A striking pattern is that population sizes for the extant species $\left(\theta_{A},\theta_{B},\theta_{C},\theta_{D}\right)$ are much better estimated, with much narrower CIs, than those for the ancestral species $\left(\theta_{r},\theta_{s},\theta_{t}\right)$ under both models MSC and MSci (Supplementary Figs. S7 and S8).

When introgression was between non-sister species (from D into C in Fig. 1b), biases in MSC estimates of population sizes were more pronounced. Introgression caused $\theta_{C}$ for the recipient population to be overestimated under the MSC (Supplementary Fig. S9). Note that the two branches *sh* and *hC* of Fig. 1b are merged into one branch, assigned the population size parameter $\theta_{C}$ under the MSC model. As the model does not account for gene flow, the extra genetic variation in species C introduced by introgression was misinterpreted as a large population size (*θ*_*C*_). The MSC model seriously overestimated $\theta_{s}$ and $\theta_{r}$, because the corresponding divergence times ($\tau_{s}$ and $\tau_{r}$) were underestimated; as discussed above, $\tau_{r}$ and $\theta_{r}$ are expected to be strongly negatively correlated, as are $\tau_{s}$ and $\theta_{s}$ (Burgess and Yang 2008). The underestimation of $\theta_{t}$ by the MSC (Fig. 3) may be to compensate the serious overestimation of $\theta_{s}$ and $\theta_{r}$. The MSci model was capable of accurately estimating all θ for contemporary and ancestral species (Fig. 3 and Supplementary Fig. S10). Similar to estimation of divergence times, the largest improvements to θ estimates under the MSci came from longer sequences rather than more individuals.

In simulations with introgression from a ghost lineage, ancestral population sizes $\left(\theta_{r},\theta_{s},\theta_{t}\right)$ were overestimated under the MSC, especially at the high introgression rate (φ = 0.2 vs. 0.05), with posterior means of $\theta_{t}\;\text{and}\;\theta_{s}$ to be almost twice the true value (Fig. 3 and Supplementary Fig. S11). While more data typically reduce uncertainty in parameter estimates when the correct model is specified, this was not the case here in the analysis under the misspecified MSC model: the HPD CIs for $\theta_{r}\;\text{and}\;\theta_{s}$ were wider for longer sequences (500 bps instead of 100), for more samples per species (10 sequences instead of 2) and at higher mutation rate (θ=0.01 instead of 0.001). The overestimation and large uncertainty of ancestral population sizes $\left(\theta_{r},\theta_{s},\theta_{t}\right)$ here are in sharp contrast with nearly unbiased estimation of the corresponding divergence times $\left(\tau_{t},\tau_{s},\tau_{r}\right)$ discussed earlier (Supplementary Fig. S5). While the divergence times were informed by the minimum between-species coalescent time or the smallest between-species sequence divergence, the ancestral population sizes are determined by the variance as well as the mean in sequence divergence among loci (Burgess and Yang 2008), and the variance is greatly inflated by the introgression from the ghost species. Population sizes for contemporary species $\left(\theta_{A},\theta_{B},\theta_{C},\theta_{D}\right)$ were less affected by the introgression and much better estimated under the MSC, with $\theta_{A}$ slightly overestimated, compensated by a slight underestimation of $\theta_{B}$ (Supplementary Fig. S11). Finally, the MSci model was capable of accurately estimating all θ parameters (Supplementary Fig. S12).

#### Introgression probabilities

When introgression was between sister species, posterior means for φ were close to the true values only in informative datasets with high mutation rate (*θ* = 0.01) and long sequences (500 bp) (Supplementary Fig. S2). In uninformative datasets of short loci (100bp) or low mutation rate (*θ* = 0.001), the posterior for φ was very close to the prior. However, when introgression was between non-sister species or involved a ghost lineage, φ was accurately estimated at both low and moderate rates (with φ=0.05 or 0.2) (Supplementary Figs. S4 and S6). Posterior means slightly overestimated φ when the sequence length was 100 bp, but the true values were well within the 95% HPD CIs. The results suggest that it is in general harder (and more informative datasets are needed) to estimate the introgression rate between sister lineages than between non-sister lineages.

### Approximating Continuous Gene Flow With MSci

#### Divergence times

**Figure 4.**
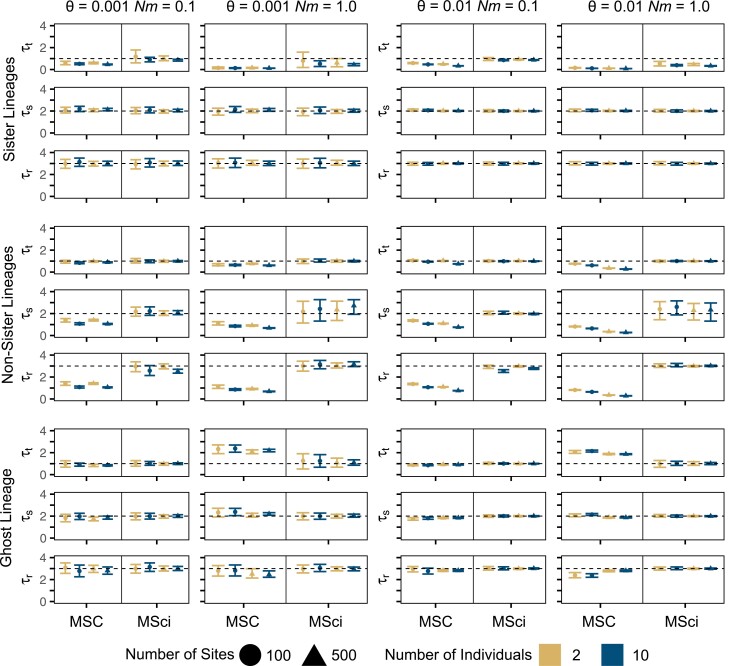
Divergence time estimates for speciation nodes simulated under the IM model. True values are shown with a dashed horizontal line. Points are posterior means and error bars are 95% HPD CIs, averaged over 10 replicates. The *y*-axis scale is $\times 10^{-3}$ and $\times 10^{-2}$ for when θ=0.001 and θ=0.01, respectively.

With continuous gene flow between sister species (from B to A, Fig. 1a) and at the low migration rate (Nm=0.1), $\tau_{t}$ was underestimated by the MSC but with very little bias under the MSci (Fig. 4 and Supplementary Fig. S13 and S14). Ages of the older nodes $\left(\tau_{s},\tau_{r}\right)$ were well estimated by both the MSC and MSci models (Fig. 4). The results are similar to those obtained from simulation under the MSci model. However, at the high migration rate (Nm=1), $\tau_{t}$ was underestimated by both the MSC and MSci although the bias was greater under the MSC (Fig. 4 and Supplementary Figs. S13 and S14). We note that Nm=1 is an unrealistically high rate for migration between species. The time of introgression is undefined when the data are generated under the continuous migration model, but the estimates were much more recent than the midpoint of the time period of gene flow (Supplementary Fig. S14). This is because the introgression time is dominated by the smallest sequence divergence between the species (A and B), generated by the most recent migration events (Huang et al. 2022b).

For gene flow between non-sister species, the MSC estimates of $\tau_{s}$ and $\tau_{r}$ almost collapsed to the same value, and both ages were severely underestimated, especially at the high migration rate (Nm=1) (Supplementary Fig. S15). MSci was able to recover the species divergence times correctly, with the exception of $\tau_{s}$, the estimate of which had wide CIs (Supplementary Fig. S16). Although the MSci model assumes episodic gene flow, it performed well when there was continuous gene flow between non-sister lineages, much better than the MSC (Fig. 4).

When continuous gene flow occurs from an unsampled outgroup species O into species A at the low rate (*Nm* = 0.1), the MSC model provided reasonable estimates of divergence times, with the true value within the HPD CIs (Fig. 4 and Supplementary Fig. S17). At the high migration rate *Nm* = 1, the MSC overestimated $\tau_{t}$. This may be due to the attempt by the MSC model to account for the large sequence distances between A and any of B, C, and D caused by gene flow. In contrast, the MSci model accurately estimated all divergence times, regardless of the migration rate or other simulation conditions (Supplementary Fig. S18).

#### Population sizes

**Figure 5.**
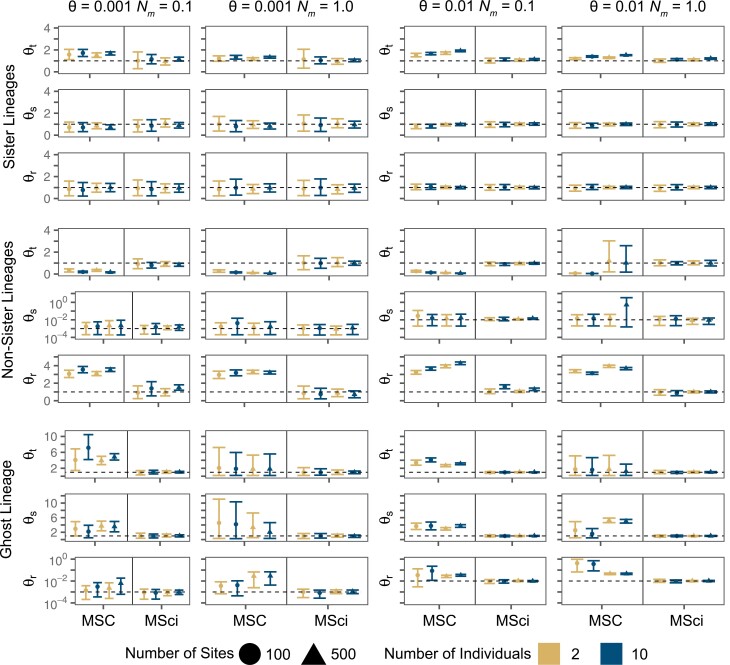
Population size estimates for speciation nodes simulated under the IM model. True values are shown with a dashed horizontal line. Points are posterior means and error bars are 95% HPD CIs, averaged over 10 replicates. $\theta_{s}$ for the non-sister scenario and $\theta_{r}$ for the ghost lineage scenario are shown on the log10 scale. The *y*-axis scale is $\times 10^{-3}\text{and }\times 10^{-2}$ for when θ=0.001 and θ=0.01, respectively.

In simulations of continuous gene flow between sister species (from B to A), the MSC model overestimated $\theta_{A}$ for the recipient species and underestimated $\theta_{B}$ and $\theta_{t}$, with more serious biases in more informative datasets (with more sites or more individuals) (Supplementary Fig. S19). The MSci model did not have such biases despite the mismatch in the assumed mode of gene flow (Fig. 5), except that $\theta_{t}$ was overestimated at the high sequence divergence (*θ* = 0.01) (Supplementary Fig. S20).

In simulations of continuous gene flow between non-sister species (from D to C; Fig. 1b), the MSC model produced biased estimates of *θ* (Fig. 5). Estimates of $\theta_{s}$ were large and implausible in some cases (note the logarithmic scale for *θ*_*s*_ in Fig. 5). Contemporary θ were affected as well (Supplementary Fig. S21). Using the MSci model to analyze data with continuous gene flow produced largely correct estimates of ancestral θ, although $\theta_{C}$ for the recipient species was overestimated and $\theta_{D}$ for the donor species underestimated at the high migration rate (*Nm* = 1) (Supplementary Fig. S22). The ancestral population size for the donor species ($\theta_{g}$) was also overestimated by the MSci.

When there was continuous gene flow from a ghost outgroup lineage (O) into species A (Fig. 1c), the MSC overestimated the population sizes for the ancestral species $\left(\theta_{r},\theta_{s},\theta_{t}\right)$; notably, $\theta_{r}$ was overestimated by two orders of magnitude at the high migration rate (*Nm* = 1) (Fig. 5). This overestimation may be explained by the inflated variance in sequence divergence between A and any of B, C, and D. Furthermore, $\theta_{A}$ for the recipient species was overestimated by the MSC, because of the extra genetic variation introduced by gene flow. Other contemporary θ were within the HPD CIs, except a few cases with short sequences (Supplementary Fig. S23). The MSci model was able to correctly estimate all population sizes despite not having data for the ghost lineage (Supplementary Fig. S24).

#### Introgression probabilities

Although φ in the MSci model may be hard to interpret when the generating model assumes continuous migration, understanding the behavior of the estimated φ under MSci from data generated under the IM model may be useful when those models are applied to the same dataset. Huang et al. (2022b) noted that the estimated φ in the MSci model is smaller than the expected proportion of immigrants, accumulated over the whole time period of gene flow:


$$\begin{array}{l} \varphi_{0}=\text{1}\text{ exp}(\text{4}M\Delta \tau /\theta ), \end{array}$$ (1)

where *M* = *Nm* is the expected number of migrants per generation, *N* is the effective population size of the recipient species, and Δ*τ* is the time duration of continuous migration. Here *φ*_0_ is the probability under the IM model that a sequence in the recipient population is traced back to the donor population, irrespective of the migration time, when one traces the genealogical history of the sample backward in time. We have *φ*_0_ = 0.33 at *Nm* = 0.1 and *φ*_0_ = 0.98 at *Nm* = 1 for the case of gene flow between sister lineages (Fig. 1a) compared with the mean estimates of 0.14 and 0.49 when θ=0.01 for 500 bp loci (Supplementary Fig. S14). For gene flow between non-sister lineages (Fig. 1b), we have *φ*_0_ = 0.55 at *Nm* = 0.1 and *φ*_0_ = 1.00 at *Nm* = 1, compared with the estimates of 0.47 and 1.0 (Supplementary Fig. S16). For Fig. 1c (migration from a ghost lineage), the expected proportion of introgression was *φ*_0_ = 0.33 at *Nm* = 0.1 and *φ*_0_ = 0.98 at *Nm* = 1, compared with the estimates of 0.37 and 0.98 (Supplementary Fig. S18). Those comparisons suggest that Equation (1) provides a useful guide for interpreting parameter estimates when both the IM and MSci models are applied to the same data. Note that if continuous migration occurs over an extended time period, even a small migration rate (*M*) in the IM model may correspond to a high introgression probability in the MSci model.

Overall, many features of the simulations under the MSci model were recapitulated in the simulations under the IM model. The MSC estimates were in general incorrect but MSci were able to recover the true divergence times if the migration rate was not extremely high.

### Analyses of Empirical Data

#### Adansonia

We used the stepping-stones approach to calculate the logarithm of the marginal likelihood for four different models, with zero, one or both introgression events: $\varphi_{w\to x}$ for introgression from mainland African *A. digitata* to the Malagasy section Brevitubae and $\;\varphi_{y\to z}$ for introgression from the early diverging *A. rubrostipa* lineage into the common ancestor of the core section Longitubae (Supplementary Fig. S25) (Karimi et al. 2020). Our marginal likelihood calculations favored the model with both introgression events (Table 1). However, the standard error (SE) for this calculation was too large to be trustworthy. We thus applied the approach of Ji et al. (2022), which calculates the Bayes factor comparing two nested models via the Savage–Dickey density ratio. Here B_21_ is the Bayes factor in support of the two-rates model against the one-rate model, with the simpler one-rate model accounting for the y→z introgression but not the w→x introgression, while the more-complex two-rates model accounting for both introgression events. In other words the Bayesian test using B_21_ tests the null hypothesis $H_{1}\text{:}\varphi_{w\to x}=0$ against the alternative hypothesis $H_{2}\text{:}\varphi_{w\to x}e0$ when the model already accommodates $\varphi_{y\to z}$. We define a null region of negligible gene flow in the alternative hypothesis, $\emptyset :\;\varphi_{w\to x}<\varepsilon$, where ε is a small constant (for which we use 0.01 and 0.001). Then B_21_ is given by the ratio of the prior and posterior probabilities for the null region under the two-rates model *H*_2_:

**Table 1.** Marginal log-likelihood values and posterior model probabilities in analyses of the two empirical datasets

Dataset	Introgression prob.	Log marginal likelihood	SE	*P* (model)
*Adansonia*	None	−1,701,076	3.35	4.9 × 10^−28^
	$\varphi_{w\to x}$	−1,701,050	3.47	9.5 × 10^−25^
	$\varphi_{y\to z}$	**−1,700,996**	**3.49**	**0.27**
	$\varphi_{w\to x},\varphi_{y\to z}$	−1,700,995	3.55	0.73
*Jaltomata*	None	−1,914,473	3.33	6.8 × 10^−46^
	$\varphi_{w\to x},\varphi_{y\to z}$	−1,914,404	3.18	6.3 × 10^−16^
	$\varphi_{w\to x},\;\varphi_{y\to z},\varphi_{u\to v}$	**−1,914,369**	**3.53**	**1**

Note: The log marginal likelihood was calculated using the stepping-stones sampling, with the standard error (SE) indicating uncertainty in the calculation. The assumed introgression probabilities in the model are defined in Supplementary Figs. S25 and S32 for the two datasets, respectively. The best model for each dataset is shown in bold.


$$\begin{array}{l} B_{21,\varepsilon \;}=\frac{\text{Pr}(\emptyset )}{\text{Pr}(\emptyset |\text{x})}=\frac{\varepsilon }{\text{Pr}(\emptyset |\text{x})}, \end{array}$$ (2)

where the prior probability is $\text{Pr}(\emptyset )=\text{Pr}\{{\varphi_{w}}_{\to x}<\varepsilon \}=\varepsilon$ as the prior is uniform ${\varphi_{w}}_{\to x}\;$ ~ U(0, 1), and Pr(∅|x) is the posterior probability, estimated by the proportion of MCMC samples in which $\varphi_{w\to x}<\varepsilon$. We obtained $B_{21}=0.0121$ for ε = 0.01 and $B_{21}=0.0043$ for ε = 0.001. Thus at the significance level of about 1%, the Bayesian test rejected the two-rate model and supported the one-rate model with section Longitubae introgression only $\left(\varphi_{y\to z}\right)$. Note that unlike frequentist test, the Bayesian test may strongly support the null hypothesis. Consistent with our test, Karimi et al. (2020) suggested that introgression between mainland African and Malagasy *Adansonia* was a technical artifact due to non-significant *D-*statistic results.

**Figure 6.**
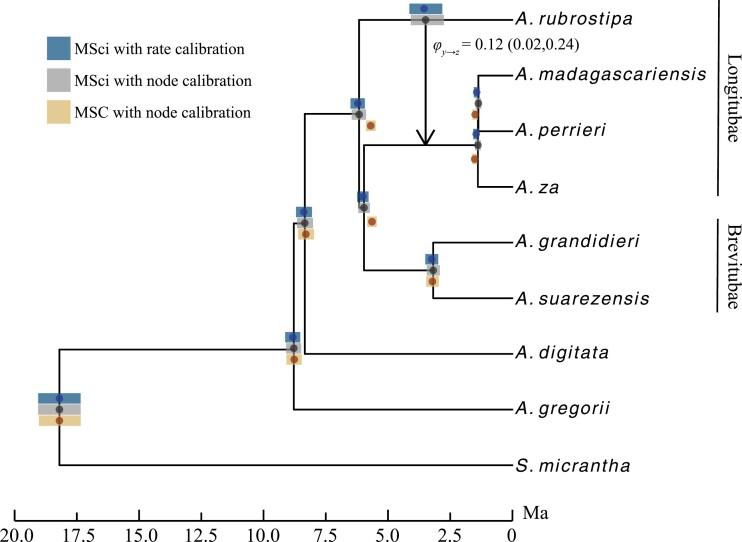
Divergence time estimates for *Adansonia*. Node heights are posterior means under the MSci with node-calibrated divergence times, indicated by the black dot. Error bars are 95% HPD CIs. The vertical line with an arrow shows the time, and direction of introgression with the posterior mean and 95% HPD CI of introgression probability displayed. The posterior mean and 95% HPD CI for the Longitubae introgression event are shown on the *A. rubrostipa* branch. Vertical bars along the right show section names based on the morphological classification of Malagasy baobabs.

The introgression probability was estimated under the one-rate MSci model to be $\varphi_{y\to z}=0.12$, with the time of introgression around 3.5 Ma ago (Fig. 6). As found in the simulation with introgression between non-sister species, the MSC model, by ignoring gene flow, led to underestimation of species divergence time (i.e., the time of origin of Malagasy baobabs) compared with the MSci analysis (Fig. 6 and Supplementary Table S2). However, the differences were not large. In all cases, the 95% HPD CIs overlapped between the MSC and MSci analyses and both implied a late Miocene origin of Malagasy baobabs, with diversifications through the Pliocene and early Pleistocene (Fig. 6).

We assessed MCMC mixing by using four replicate runs for the same analysis. All divergence time and population size parameters for *Adansonia* speciation nodes under the MSC and MSci converged with consistent unimodal distributions (Supplementary Figs. S25–S29). However, some parameters around the Longitubae introgression event under the MSci model $\left(\tau_{z}\text{,}\;\theta_{z}\;\text{and}\;\varphi_{y\to z}\right)$ showed bimodal posteriors, with strong correlations between parameters. We used the MCMC samples from the runs that did not show mixing problems to generate posterior summaries.

#### Jaltomata

The ASTRAL species tree constructed using ML gene trees was identical to the concatenated ML tree in the original study (Wu et al. 2018) except for the placement of *J. dendroidea* and *J. incahuasina*, which had a low local posterior probability (Supplementary Fig. S30). PhyloNetworks inferred three introgression events based on slope heuristics. The *major species tree* implied by the network, formed by taking the parental branch with the larger admixture proportion at each hybridization node, was identical to the ASTRAL species tree. SNaQ recovered two introgression events between sampled lineages that were restricted within the purple-fruited clade, and between the green- and orange-fruited clades (Supplementary Figs. S31 and S32). SNaQ also recovered an introgression event between an unsampled lineage that diverged prior to the MRCA of *Jaltomata* and the ancestor of the green- and orange-fruited clades. This *Jaltomata* ghost lineage hypothesis was supported by our marginal likelihood calculation (Table 1). The original study (Wu et al. 2018) calculated multiple significant *D-*statistics indicating ancient introgression events between the purple-and orange-fruited clades, which may be related to the introgression involving the ghost lineage detected here with SNaQ. The inferred introgression events were used to estimate divergence times and population sizes under the MSci model while the major species tree was assumed with the MSC (Supplementary Fig. S32).

**Figure 7.**
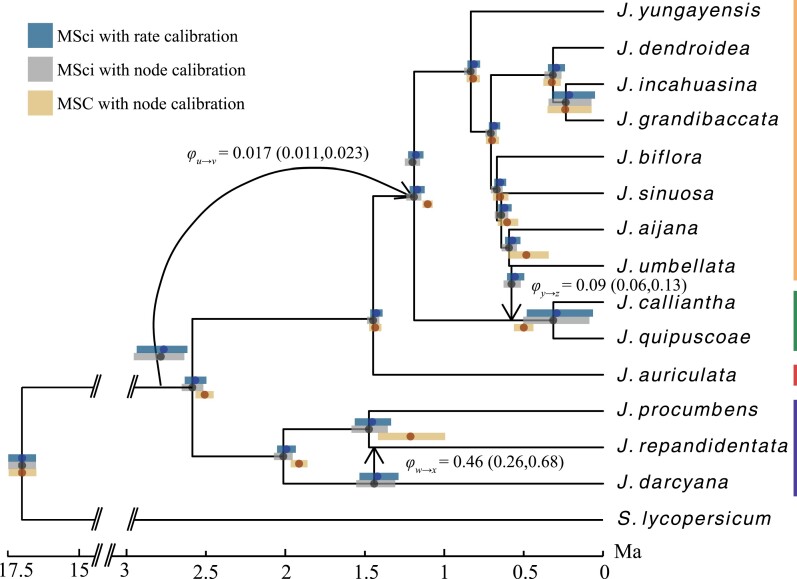
Divergence time estimates for *Jaltomata*. node heights are posterior means under the MSci with node-calibrated divergence times. Reticulate edges are shown by black arrows along with their corresponding introgression probabilities from the MSci model. Posterior means and 95% HPD CIs for the ghost introgression donor and recipient nodes are shown above their vertices to improve visibility. Vertical bars along the right show fruit colors among lineages.

Results obtained under the MSci and MSC models (Supplementary Table S3) largely agree with patterns observed in simulations. Divergence times between species involved in gene flow were more recent with the MSC compared with the MSci. For example, introgression between *J. darcyana* and *J. repiandentata*$\left(\varphi_{w\to x}\right)$</mathgraphic> led to an MSC estimate of more recent divergence time between those species than under the MSci, although the 95% HPD CIs overlapped across calibration strategies (Fig. 7). Similarly, introgression from the orange-fruited *J. umbellata* to the green-fruited clade $\left(\varphi_{y\to z}\right)$ led to a notably younger estimate of the age of their common ancestor under the MSC. The ancient introgression from an unsampled lineage had very little effect on divergence times in the *Jaltomata* analyses, presumably because the estimated introgression probability $\left(\varphi_{u\to v}\right)$ was very low (Fig. 7). Overall, the MSC and MSci estimates of divergence times are similar, and absolute dates under both models are consistent with the original concatenated ML estimates (Wu et al. 2018).

Including introgression events in the MSci model introduces considerable model complexity, but it is possible to obtain convergence and well-sampled posteriors from empirical datasets such as the one from *Jaltomata*. The θandτ parameters converged across all nodes for both the MSC (Supplementary Figs. S33 and S34) and MSci analyses (Supplementary Figs. S35 and S36). The only parameters that had inefficient MCMC sampling in the MSci analyses were those corresponding to the ghost introgression event $\left(\theta_{u},\theta_{v},\tau_{u},\tau_{v}\right)$ and the age of the orange-fruited clade $\left(\tau_{f}\right)$.

## Discussion

### Introgression Can Mislead Divergence Time Estimation

The impact of gene flow on divergence time estimation is expected to be complex, depending on the rate of gene flow, the species involved in the gene flow, and the relative position of the nodes on the species tree we are attempting to date (e.g., whether they are ancestral to or descendants of the hybridization node). We note that species divergence times are often well constrained and well estimated with narrow CIs, and tend to be influenced by the smallest sequence divergence between the species. Thus when gene flow is present but ignored by the MSC model, the divergence time between the species involved in gene flow is underestimated. Bias in divergence times for other nodes on the species tree are harder to predict, as they depend on the position of the nodes relative to the gene-flow events. In contrast to species divergence times, ancestral population sizes are often estimated with large uncertainty and the estimates are easily affected by model misspecification. Thus the impact of gene flow is expected to be far greater on ancestral population sizes than on species divergence times.

Our simulations demonstrate that even a low level of introgression can cause underestimation of divergence times when it is not accounted for in the MSC model. Previous studies showed that when gene flow between sister species is ignored, the age of their common ancestor will be underestimated (Leache et al. 2014; Barley et al. 2018). Our simulations of introgression between sister species show that the divergence time of the sister species is underestimated by the MSC, consistent with previous studies, but notably, there is little error in the estimated ages for other nodes (Fig. 2). We also show similar underestimation under the MSC model of divergence times between non-sister species that experienced introgression (Fig. 2) and that the bias becomes more serious in more informative datasets, with more individuals or longer sequences. With continuous migration over time between non-sister species, the MSC underestimates not only the time of divergence between the two species involved in gene flow ($\tau_{r}$ in Fig. 4), but also the ages of the descendent nodes ($\tau_{s}$ and $\tau_{t}$ in Fig. 4 and Supplementary Fig. S15). By explicitly modeling introgression events in the MSci model, accurate estimation of species divergence times (Fig. 2), as well as the time of introgression (Fig. 1), can be achieved.

When gene flow occurs continuously over an extended time period, as assumed in the IM model, both the migration rate (*m* or proportion of immigrants per generation) and the time duration of gene flow are important. Gene flow occurring at a low rate over a long time period may be as influential as gene flow at a high rate over a short time period. Continuous gene flow had effects similar to episodic introgression, causing the MSC model to underestimate the divergence time between the species involved in gene flow. In our simulations with gene flow from a ghost lineage, the effects on divergence time estimation was noted to depend on whether gene flow was episodic or continuous. Under episodic introgression (as in the MSci model), the MSC only slightly underestimated the age of the parent node of the lineage-receiving genes (Fig. 2). However, continuous gene flow caused species divergence times to be *over*-estimated under the MSC ($\tau_{s}$ and $\tau_{t}$ in Fig. 4 and Supplementary Fig. S17), apparently because the excessive amount of gene flow inflated the between-species sequence distances. The MSci model is capable of correctly estimating all divergence times in the presence of a ghost lineage, regardless of whether gene flow is episodic or continuous (Figs. 2 and 4). The effects of unsampled lineages are particularly concerning as extinct lineages are generally unobserved and may go unaccounted for in many studies except in the case of multiple lines of evidence or fossils that point toward their existence (e.g., Hey et al. 2018). When *a priori* information about a ghost lineage or sampling gaps is available, the MSci model can accurately estimate divergence times despite the absence of data from the ghost lineage, as expected of coalescent estimators (Beerli 2004), even if episodic introgression is a simplifying assumption about a more-complex pattern of gene flow (Fig. 4 and Supplementary Fig. S18). However, this conclusion may be too optimistic. In analysis of empirical data, it may be challenging to infer introgression events involving ghost lineages. The molecular clock assumption may be of concern as well, if life history traits (such as selfing and generation time) differ among species.

### Population Sizes: A Valuable Nuisance

We also compared the performance of the MSC and MSci models for estimating θ when there is gene flow between species. Although θ is a nuisance parameter when our interest is in divergence times, it is nonetheless valuable for providing estimates of effective population sizes (e.g., Poelstra et al. 2021). In general, θ is easier to estimate for contemporary species than for ancestral species. When there is gene flow (either episodic introgression or continuous migration), the MSC model may produce biased estimates of θ. In the case of introgression between sister and non-sister species, MSC overestimates θ for the common ancestor of the two species involved in gene flow while underestimating their divergence time (Figs. 3 and 4).

The MSci model is able to correct for these biases when MSci was the generating model (Fig. 4). When the data are generated under the IM model and the migration rate is high, however, the misspecification of the mode of gene flow may cause MSci to produce biased estimates of θ, in particular, if gene flow is between non-sister species or from a ghost lineage. If gene flow is suspected to occur over extended periods of time, the IM model may be more realistic than the MSci model.

### Introgression Probability and the Mode of Gene Flow

The introgression probability was correctly estimated when data were simulated under the MSci model for all three scenarios as long as the data are informative (e.g., with long sequences and high mutation rates) (Supplementary Figs. S2, S4 and S6). If gene flow is continuous, the estimates of *φ* may be hard to interpret. We note that even seemingly low migration rates may generate *φ* estimates close to 100% if migration has occurred over an extended time period.

Furthermore, we caution against using *φ* estimates under the MSci model to infer the mechanism of speciation; *φ* values close to 0.5 are not evidence for hybrid speciation since alternative continuous migration rates and scenarios could result in such estimates. Note that when the MSci model is applied to analyze genomic sequence data, the *φ* parameter reflects the combined effects of gene flow and natural selection which purges introgressed alleles. As a result, *φ* is expected to vary across the genome and over time.

### More Individuals or Longer Sequences?

Optimizing study design has been a focus of previous investigations. For example, Felsenstein (2006) demonstrated that adding sites in the sequence can be an inefficient approach to improving the estimation of *θ* for a single population. A noteworthy result from our simulation is that sampling more individuals per species had only limited effects. Ten individuals provided little improvement in the accuracy of the posterior estimates of either *τ* or *θ* in all simulation scenarios. More individuals did reduce HPD CIs under the MSci but also increased the systematic bias for affected nodes under the misspecified MSC model when there was introgession between non-sister species (Supplementary Figs. S3, S7, S15, and S21). Thus, while the number of individuals may be important for dating population or species divergences with population genomic methods based on the site frequency spectrum (Gutenkunst et al. 2009; Excoffier et al. 2013), the MSci model can accurately estimate divergence and population size parameters with a single diploid individual (or two haploid sequences). Our simulation suggested that instead of additional individuals, it may be more beneficial to obtain longer or more variable loci.

We note that enriched libraries are used to extract different parts of the genome such as ddRAD-seq, ultra-conserved elements, transcriptomes and exomes (Faircloth et al. 2012; Breinholt et al. 2018; Tagliacollo and Lanfear 2018). Some of those markers are highly conserved (such as exons) while others are more variable. As a result, the number of variable sites per alignment may differ markedly among those different types of data, so that the amount of information available to MSC-based analyses also varies.

Huang et al. (2020) conducted a simulation study to examine the impacts of various factors on analyses under the MSC model with and without gene flow, such as the number of loci, the number of sampled sequences per species, the sequence length, and the mutation rate. Overall the number of loci was found to be the most important factor, while the number of individuals sampled per species is the least influential (Huang et al. 2020, Table 6). Our results are consistent with those results although in our simulation the number of loci was fixed at 1000 loci.

### Biological Interpretations of Slight Estimation Biases

Our reanalysis of the *Adansonia* data suggested relatively small effects of introgression on divergence time estimation. The introgression rate within section Longitubae ($\varphi_{y\to z}$Fig. 6) was estimated to be 12%, and the MRCA of *A. rubrostipa* and the core Longitubae (*A. za*, *A. perrieri*, and *A. madagascariensis*) was only 7% younger in the MSC estimate compared to MSci. Our simulations of non-sister lineage introgression under the MSci model suggested stronger effects, such that introgression probabilities of 0.05 and 0.2 caused the MSC to underestimate the MRCA $\left(\tau_{r}\right)$ true divergence time by approximately 15% and 50%, respectively (Fig. 2 and Supplementary Fig. S5). In the empirical dataset the species tree is larger with more tips and speciation events, and the denser taxon sampling may have caused the node ages to be less affected by introgression thus limiting the estimation bias. The MSC model resulted in a slightly younger estimate of the age of Malagasy *Adansonia*, but both the MSC and MSci estimates suggest late Miocene origins, with a difference of about 450 Ka. This difference is of little consequence concerning the divergence time. However, studies of Pleistocene climatic oscillations could be greatly misled by errors of a few hundred Ka.

Similarly, the *Jaltomata* transcriptome analyses highlighted some expectations from our simulations. For example, the age of the purple-fruited clade was younger in the MSC analyses relative to estimates under the MSci with introgression from *J. darcyana* to *J. repandidentata* ($\varphi_{w\to x}$, Fig. 7). This difference was the largest observed in our empirical analyses, apparently because of the high introgression probability $\left(\varphi_{w\to x}=0.46\right)$</mathgraphic>, although the HPD CIs overlapped between models. In the case of introgression between the orange-fruited *J. umbellata* and the green-fruited common ancestor ($\varphi_{y\to z},\;$Fig. 7), the divergence between *J. calliantha* and *J. quipuscoae* was older in the MSC analyses compared with the MSci, because the time of introgression was older than speciation, and the bias observed here was not covered in our simulations. This introgression event is also associated with more recent MSC estimates of divergence between *J. umbellata* and *J. aijana*, and the ancestor of the green- and orange-fruited clades, as anticipated from simulations, but the differences between all mean posterior estimates are typically small. The age estimates regardless of model and calibration choice would all imply that *Jaltomata* diversified through the Pleistocene. Although we tested the presence of a ghost lineage with marginal likelihood analyses (Table 1) and modeled it in the MSci, the effect on divergence times was negligible. The *Jaltomata* clade was younger rather than older in the MSC analysis and was contained within the MSci 95% HPD CI. This is likely due to the very small introgression probability estimated here ($\varphi_{u\to v}=0.017,$Fig. 7). The MSC was also robust to continuous gene flow for *Nm* = 0.1 in our simulations (Fig. 4), suggesting our *Jaltomata* estimates are reasonable. Although ghost lineages can be important for explaining the evolutionary history of some groups, especially in cases of adaptive introgression, their effects on divergence times are likely small unless a large number of genes were retained from the introgression event. Ultimately, our MSci analysis of *Jaltomata* agrees with the original study that used concatenation and ML, suggesting that at least some divergence time estimates are robust to both the effects of ILS and introgression.

### Implications to Real Data Analysis

Many approaches are available for detecting introgression from phylogenomic data and several allow estimation of divergence times and population sizes as well as the rate of gene flow under either continuous (Groneau et al. 2011; Hey et al. 2018) or episodic gene flow (Wen et al. 2016; Zhang et al. 2018a; Flouri et al. 2020). Here, we showed how divergence time estimation can be biased when gene flow is present but ignored in the MSC model, and that an episodic model can perform well regardless of the mode of gene flow. Consistent with previous simulations (Huang et al. 2020), the precision and accuracy of divergence time estimation critically depend on the size and information content of the dataset. The informative datasets generated in our simulation, with 1000 loci of long sequences (500 bp), with 10 sequences sampled per species per locus, and at the high mutation rate (θ= 0.01), appeared sufficient to accurately estimate model parameters under MSci. However, estimates may involve large uncertainties in the less-informative datasets.

In empirical studies, the mutation rate may be influenced by the choice of the genomic fragments targeted by the sequencing technology; for example, ultraconserved elements (UCEs) may have a lower rate than generic noncoding regions, while exons may have the lowest rate. The length of the sequenced fragments may also depend on the sequencing technology. When whole-genome sequences are available, short segments that are far apart can be extracted and then analyzed under coalescent models as independent loci, in which case the length of each locus may depend on the genetic diversity in the group (e.g., Thawornwattana et al. 2022; Zhu et al. 2022). One advantage of the MSci model, based on this and previous (Huang et al. 2020) simulations, over methods based on site frequency spectra (e.g., Gutenkunst et al. 2009; Excoffier et al. 2013) is that two sampled haplotypes (i.e., one diploid individual) should be sufficiently informative for parameter estimation. Thus, MSci analyses could accommodate many types of sequencing and sampling strategies, with the lower limit set by the requirement for informative loci and the upper limit set by available computational resources.

In our simulations, the MSC model ignoring gene flow produced reliable divergence time estimates in some scenarios but strongly biased estimates in others. Overall, the impact is greater when gene flow occurs at high rates (e.g., *φ* = 0.2 versus 0.01 in the MSci model or *M* = *Nm* = 1 versus 0.1 in the IM model), when the dataset is large and informative, and when speciation nodes are close to lineages involved in gene flow on the species tree. It is currently hard to predict whether the MSC estimates are robust to gene flow, and analyzing the data using both the MSC and MSci models appears to be the only prudent approach. One strategy may be to analyze a subset of the data under both the MSC and MSci models to test for gene flow and to assess its impact on divergence time estimation.

## Supplementary Material

Data available from the Dryad Digital Repository: https://doi.org/10.5061/dryad.zs7h44j8x.

## Data Availability

Scripts and bpp control files for simulation, plotting, and empirical data analyses are available through the Dryad Digital Repository: https://doi.org/10.5061/dryad.zs7h44j8x. Materials for simulation are also available on GPT’s GitHub page: https://github.com/gtiley/bpp_simulationTools.
